# Supplementary material for: Dietary Fibre Modulates Gut Microbiota in Late Pregnancy Without Altering SCFA Levels, and Propionate Treatement Has No Effect on Placental Explant Function
Source: Nutrients. 2025 Apr 1;17(7):1234. doi: 10.3390/nu17071234 (PMC11990268; doi:10.3390/nu17071234)
Supplement: Supplementary file 1 [file nutrients-17-01234-s001.zip › nutrients-3529683-supplementary.pdf]

Table S1. Primers used to validate bacterial abundance in faecal speciments.

Bacteria Genus Name	Primer Sequence (5′-3′)
Bilophila	F: CGCCGGTATCGAAATCGTGA<br>R: ATTCGCGGAAGGAGCGAGAG
Sutterella	F: CGCGAAAAACCTTACCTAGCC<br>R: GACGTGTGAGGCCCTAGCC
Odoribacter	F: TGTAATGATGAGCACTCTAACGG R: GGCTTTTGAGATTGGCATCC
Faecalibacterium	F: CCCCTACATCCGCTTCGACT<br>R: CTCATCACCTTCCTCCGGCT
**Total Bacteria (16S RRNA GENE)**	F: GCAGGCCTAACACATG CAAGTC<br>R: CTGCTGCCTCCCGTAGGAGT.

Table S2. Primers used to examine Mitochondrial Content and Gene Expression in placental tissue treated with short-chain fatty acids.

Primer Name	Primer Marker	Primer Sequence (5′-3′)
**MITOCHONDRIAL CONTENT**		
**MTRT4**	Mitochondrial DNA	F: ATGGCCCACCATAATTACCC<br>R: CATTTTGGTTCTCAGGGTTTG
**MTRT5**	Mitochondrial DNA	F: GCCTTCCCCCGTAAATGATA<br>R: TTATGCGATTACCGGGCTCT
**MTAIB**	Nuclear DNA - Housekeeper	F: GAGTTTCCTGGACAAATGAG<br>R: CATTGTTTCATATCTCTGGCG
**MTBA**	Nuclear DNA - Housekeeper	F: AGCGGGAAATCGTGCGTGAC<br>R: AGGCAGCTCGTAGCTCTTCTC
**GENE EXPRESSION**		
**SOD1**	Antioxidant	F: GAGCAGAAGGAAAGTAATGG<br>R: GATTSSSGTGAGGACCTGC
**SOD2**	Antioxidant	F: ATCATACCCTAATGATCCCAG<br>R: AGGACCTTATAGGGTTTTCAG
**CAT**	Antioxidant	F: AGAGAAATCCTCAGACACATC<br>R: CAGCTTGAAAGTATGTGATCC
**CASP3**	Apoptosis	F: AAAGCACTGGAATGACATC<br>R: CGCATCAATTCCACAATTTC
**CASP8**	Apoptosis	F: CTACAGGGTCATGCTCTATC<br>R: CTACAGGGTCATGCTCTATC
**TBP**	Housekeeper	F: GCCAAGAGTGAAGAACAG<br>R: GAAGTCCAAGAACTTAGCTG
**ACTB**	Housekeeper	F: GACGACATGGAGAAAATCTG<br>R: ATGATCTGGGTCATCTTCTC

Table S3. Maternal information and pregnancy outcomes from women who donated their placentae for use in tissue explants ($n = 4$).

Maternal Data	*Ethnicity (Caucasian%)*	*50%*
	Pre-Pregnancy BMI (kg/m^2)	37.13±8.532
	38-Week BMI (kg/m^2)	39.52±8.048
	Age (Years)	35.75±4.008
Pregnancy Outcomes	Offspring Sex (Male%)	50%
	Birthweight (g)	3277±196.1
	Delivery (Vaginal%)	0%
	Placental Weight (g)	776.4±108.5

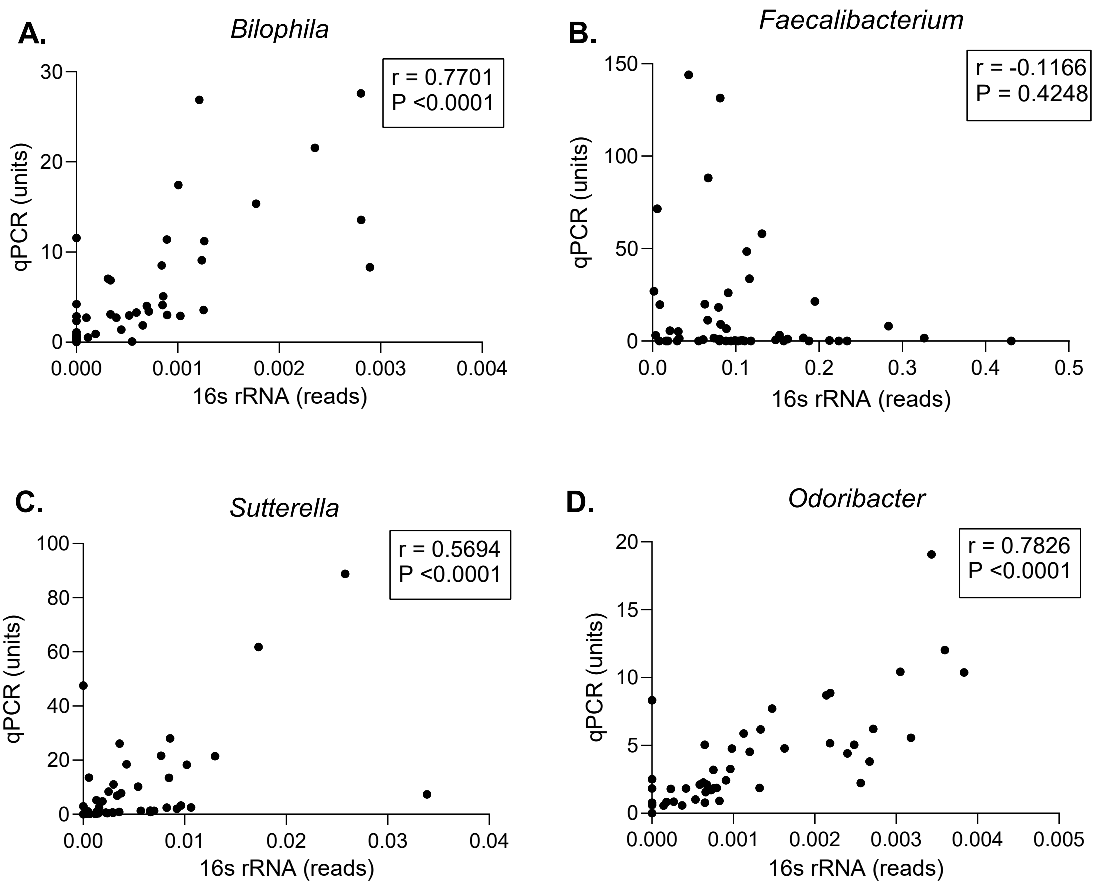

Figure S1. Correlations between qPCR assessment of abundance and normalised 16S rRNA gene amplicon reads for genera A. Bilophila, *B. faecalibacterium*, *C. sutterella*, and *D. odoribacter*. Statis-tical analysis is Individual dots represent a single sample ($n = 49$). Statistical analysis Spearman's correlation with coefficient (r) and p-value presented above. Significance accepted $p < 0.05$. Data analysed using GraphPad Prism 8.
